# 10-year longitudinal study of malaria in children: Insights into acquisition and maintenance of naturally acquired immunity

**DOI:** 10.12688/wellcomeopenres.16562.1

**Published:** 2021-04-09

**Authors:** John W.G. Addy, Yaw Bediako, Francis M. Ndungu, John Joseph Valetta, Adam J. Reid, Jedida Mwacharo, Joyce Mwongeli Ngoi, Joshua Wambua, Edward Otieno, Jennifer Musyoki, Khadija Said, Matthew Berriman, Kevin Marsh, Philip Bejon, Mario Recker, Jean Langhorne

**Affiliations:** 1 Malaria Immunology Laboratory, Francis Crick Institute, London, UK; 2 West African Centre for Cell Biology of Infectious Pathogens, University of Ghana, Accra, Ghana; 3 KEMRI/Wellcome Trust Research Programme, Kilifi, Kenya; 4 School of Mathematics and Statistics, University of St Andrews, St Andrews, UK; 5 Parasite Genomics, Wellcome Sanger Institute, Hixton, UK; 6 Centre for Tropical Medicine and Global Health, University of Oxford, Oxford, UK; 7 Centre for Ecology and Conservation, University of Exeter, Penryn Campus, Penryn, UK

**Keywords:** Plasmodium falciparum, Clinical Malaria, Protective Immunity, Longitudinal Cohorts, Growth Curves

## Abstract

**Background:** Studies of long-term malaria cohorts have provided essential insights into how *Plasmodium falciparum *interacts with humans, and influences the development of antimalarial immunity. Immunity to malaria is acquired gradually after multiple infections, some of which present with clinical symptoms. However, there is considerable variation in the number of clinical episodes experienced by children of the same age within the same cohort. Understanding this variation in clinical symptoms and how it relates to the development of naturally acquired immunity is crucial in identifying how and when some children stop experiencing further malaria episodes. Where variability in clinical episodes may result from different rates of acquisition of immunity, or from variable exposure to the parasite.

**Methods:** Using data from a longitudinal cohort of children residing in an area of moderate *P. falciparum* transmission in Kilifi district, Kenya, we fitted cumulative episode curves as monotonic-increasing splines, to 56 children under surveillance for malaria from the age of 5 to 15.

**Results: **There was large variability in the accumulation of numbers of clinical malaria episodes experienced by the children, despite being of similar age and living in the same general location. One group of children from a particular sub-region of the cohort stopped accumulating clinical malaria episodes earlier than other children in the study. Despite lack of further clinical episodes of malaria, these children had higher asymptomatic parasite densities and higher antibody titres to a panel of *P. falciparum* blood-stage antigens.

**Conclusions:** This suggests development of clinical immunity rather than lack of exposure to the parasite, and supports the view that this immunity to malaria disease is maintained by a greater exposure to *P. falciparum*, and thus higher parasite burdens. Our study illustrates the complexity of anti-malaria immunity and underscores the need for analyses which can sufficiently reflect the heterogeneity within endemic populations.

## Introduction

Malaria is a major global health problem responsible for millions of clinical cases each year with the highest burden of mortality in children under 5 years of age ^1^. A malaria infection is caused by the protozoan parasite *Plasmodium*, with the most virulent human parasite, *Plasmodium falciparum* ( *Pf*), responsible for over 90% of malaria-related morbidity and mortality, mostly in sub-Saharan Africa ^1^. Subsequent repeated exposure to *Pf* infections eventually leads to the development of partial immunity ^2– 4^. Evidence for such immunity includes the age-associated decrease in frequency and severity of clinical malaria episodes among children living in endemic areas where *Pf* infections in older children present with lower parasite densities, infrequent malaria symptoms and may produce more *Pf*-specific antibodies ^5^.

Although repeated clinical episodes of malaria have been shown to lead to substantial and diverse host immune responses ^6^ the precise mechanism(s) by which partial immunity to malaria develops and is maintained, remains unclear. Development of partial immunity to malaria likely involves a complex interplay between an antigenically diverse parasite and a dynamic host immune response. Investigating this process within human populations is challenging given the many factors that influence the development and maintenance of immunity to *Pf* including age ^2^, genetics, the number of previous clinical episodes ^6^ as well as past and current exposure ^7^ to the parasite. While some of these factors are relatively easily quantified, accurately estimating total exposure is extremely difficult as not all exposure results in clinical manifestations. Exposure to *Pf* has been demonstrated to be extremely heterogeneous, exhibiting both temporal (seasonal) and micro-geographic variation ^7– 9^.

Longitudinal study cohorts, often considered the “goldstandard’ in observational studies of natural infection, can provide very useful insights into the development of antimalarial immunity ^10^. Individuals typically under active surveillance are followed for a number of years, during which time all clinical cases of malaria are recorded. Given the impracticality of large, continuous entomological surveys, such studies typically estimate parasite exposure based on the incidence of clinical malaria within a specified geographic area ^11^. The aggregate number of episodes an individual experiences is dependent on both the extent of their exposure to the *Pf* parasites and their level of immunity. As such, in areas with reasonably high transmission intensity, the number of episodes an individual experiences would be expected to decline over time, not necessarily because transmission intensity in that geographic area is reducing, but rather because of the development of partial immunity.

After following 56 individuals over ten-years from a longitudinal study cohort, we are able to compare the rate at which each individual acquires episodes over time, an approach only possible with long-term surveillance data-sets. In such an approach, the development of immunity against malaria may be illustrated as a cumulative malaria episode curve (previously used to study the rate of growth in young children ^12^, where a plateau in accumulated episodes from children in an endemic region may be considered as evidence of the development of immunity. By visualizing the rate of accumulation of clinical episodes for each child individually, we are better able to capture the heterogeneity of clinical episodes within the population. For a subset of individuals who stop accumulating more episodes within this age-span, we compared the levels of antibodies to selected *Pf*-antigens to help determine if the decline in the rate of accumulating episodes is related to acquisition of immunity or rather reduced exposure to the parasite.

## Methods

### Ethics and consent

The study protocol and its subsequent amendments received ethical and scientific approval from the Kenyan Medical Research Institute National Ethics Committee (KEMRI SSC 1131 & KEMRI SERU 3149). Written informed consent in the local languages (Swahili or Giriama) was required from parents/guardians for participation.

### Study population

The study took place at the KEMRI-Wellcome Trust Research Programme (KWTRP) situated next to the Kilifi County Hospital, Kilifi, Kenya. The hospital serves approximately 500,000 people living in Kilifi County. The children investigated were residents of Junju a community on the southern side of an Indian Ocean creek and inhabited by predominantly Mijikenda people. Over the last 15 years, there has been a gradual, heterogeneous decline in malaria transmission in Kilifi County ^13, 14^ whereby transmission in Junju village has remained stable with a parasite prevalence of 30% ^15, 16^ during the dry season. However, there are two high malaria transmissions seasons, May to August and October to December, during which parasite prevalence rises beyond 70%. Children are recruited into the cohort at or shortly after birth and actively monitored on a weekly basis for detection of malaria episodes until 15 years of age. Extensive and detailed records of the number and dates of malaria episodes for each child over the period they are enrolled in the cohort are maintained.

For these analyses, 56 children who were born between 2001 and 2003 were selected to determine whether there is heterogeneity in the rate of accumulation of clinical episodes with age. The cohort was started in 2001 and has recruited children every year since in the first year of life, and discontinued follow up for children reaching 15 years of age. The size of the cohort at any one point is 300–400 children. At this time, 56 children had completed 10 years of follow up and this analysis focuses on these 56 children.

A clinical malaria episode was defined as a body temperature greater than 37.5°C and 2500 parasites per microlitre of blood ^17^. A year was defined from 1st of April to the 31st of March, capturing the total number of episodes before the wet season, which normally starts in April after a relative dry period of at least four months with minimal *Pf* transmission. For example, 2015 corresponds to the 1st of April 2014 to the 31st of March 2015. Parasite load (determined by microscopy and PCR) and serum antibody levels were measured from blood samples collected at the end of the dry season each year.

### Sample collection

*Pf* episodes are normally diagnosed during weekly active surveillance carried out by a field worker based in the same village as the child. During these visits auxiliary body temperature, and or recent history of fever is taken, and if a child is febrile a blood sample is taken for a *Pf* specific rapid diagnostic test (RDT) and for blood smears. The blood smears are read later to determine the *Pf* parasite densities used in this paper, whilst immediate antimalarial treatments are administered on the basis of the RDT testing.

Additionally, an annual cross-sectional survey is conducted in March, just before the beginning of the rains that marks the beginning of the main malaria transmission season in Kilifi. During these surveys, 5ml of venous blood (for immunological studies) and blood smears for detection and subsequent calculation of the associated cross-sectional *Pf* densities and prevalences. Furthermore, q-rtPCR has been applied to all the samples collected since 2007 to complement the microscopy data.

### Determination of parasite density

Thick and thin blood films were stained with Giemsa and *Pf*-infected red cells counted against 500 leukocytes and 1,000 red blood cells, respectively. To detect lower parasite densities, a highly sensitive *Pf* -specific PCR assay based on 18 was performed.

A sensitive high qPCR assay was used for detection where 500 µl of whole venous blood was used to extract DNA using an automated DNA extraction and purification method (QIAsymphony platform, Qiagen, Germany) according to the manufacturer’s instructions. DNA was eluted in 100 µl of DNAse free water/elution buffer from which 13.5 µl was used to amplify the 18S ribosomal RNA gene by qPCR (we used Applied Biosystems’ TaqMan™Universal PCR Master Mix (cat no 4318157) which already contains the DNA polymerase (AmpliTaq Gold™DNA Polymerase)) in triplicates in a hydrolysis probe assay using primers and probes previously described. The PCR cycling conditions were carried as described using Applied Biosystems 7500 real-time PCR system. Non-template control was used as a negative control (in triplicate wells) with parasite quantification against known cultured parasite standards comprising of six serial dilutions of extracted DNA also run-in triplicate.

### Antigens for ELISA

*Pf* -specific plasma IgG plasma antibody responses were quantified against recombinant *Pf* AMA1 (FVO, 3D7 and L32 alleles), MSP1-42 kDa (3D7 and FUP allelles) and MSP3, to which circulating IgG antibodies were associated with clinical protection in previous studies ^19– 22^. Recombinant *Pf* antigens were kindly provided by L.H. Miller and colleagues from the Laboratory of Malaria and Vector Research (National Institute of Allergy and Infectious Disease, NIH, Rockville, MD, USA).

### ELISA

Plasma samples from the cross-sectional surveys of 2015, 2016 and 2017 were tested for human IgG antibodies specific for AMA1, MSP1-42 and MSP3 antigens using a standard ELISA protocol. Plasma samples were tested for human IgG antibodies specific for Pf AMA1, MSP142 and MSP3 antigens using a standard ELISA protocol. Recombinant Pf antigens were provided by L. H. Miller (National Institutes of Health, Rockville, MD). For AMA1, ELISA plates were coated with a 1:1 mixture of FVO and 3D7 alleles. Plates were coated overnight at 4 °C, with recombinant proteins at 1 *µ*g/mL in bicarbonate buffer (100 *µ*L/well). One-hundred microliters per well of 1 in 1,000 dilution of test plasma in 0.3% (vol/vol) PBST + EDTA was added after plates had been washed three times with 0.05% (vol/vol) Tween in phosphate buffered saline (PBST), and thereafter blocked with 10% (vol/vol) foetal calf serum (FCS)/PBS (200 *µ*L/well). Plates with test plasma were then incubated for 1.5 h at room temperature in a humidified chamber. Plates were then washed five times before the addition of alkaline phosphatase (AP)-labelled goat anti-human IgG Abs (Sigma) conjugate at 1:2,000 dilution 0.05% PBST at 100 *µ*L/well. After 1h incubation with the conjugate, the plates were washed five times and the human IgG complexed with the AP-labelled conjugate revealed with and P-nitrophenyl phosphate (Sigma). The substrate reaction was stopped with 50 *µ*L/well of 3 M NaOH, after which the plates were left for 5 min in the dark before being read at 405/570 nm. Antibody levels were quantified against respective standard curves on each plate of a purified hyperimmune IgG from immune adults and expressed in arbitrary units.”.

### Monotontic increasing functions

Spline functions ^23^ were fitted to the 56 children who completed the cohort study from Junju, from the age of 5 to 15. The functional relationship of accumulated malaria episodes over time *t*, *g*( *t*), may be represented as a smoothed function through linear combinations of model coefficients *c _k_* and basis functions *ϕ _k_* ( *t*), where

$$g\left(t\right)=\sum_{k=1}^{k}\left(c_{k}\phi_{k}\left(t\right)\right).$$

Shape constrained additive models were used to ensure the accumulated malaria episode function never decreased and followed a monotonic functional relationship with time ^24^. These functions were fitted in R using the SCAM package ^25^. A log-link function was used to model the malaria count data. The smoothing parameter of each SCAM was fixed at 0.01 at 7 basis functions to make lines across all children comparable. The first derivative of the fitted accumulation of malaria episodes ( *g′*( *t*)) represents the estimated number of episodes for that time point, *t*. Children who stop experiencing episodes in their last three years in the study were considered plateauers and their parasite density and antibody levels were investigated to see if this was due to a drop in exposure.

### Statistical analysis

To understand why those children experiencing no more clinical malaria episodes, measurements of the levels of AMA1, MSP1 and MSP3-specific antibodies were compared between plateauers and children who experienced episodes up to the last three years of the cohort study. Antibody measurements were measured from samples taken in 2015, 2016 and 2017 and followed a crossed design structure fitted through a mixed model framework in the R package lme4 ^26^.

Y=Xβ+Zυ+ε

Where, *Xβ* are the models fixed effects of Group (whether they plateaued in clinical episodes by the age of 12 or did not) and Year (2015, 2016 and 2017) and *Zυ* is the random effect of Participant. An F-test was used to determine the significant differences of the fixed effects based on the Kenward-Roger method ^27^ from the lmerTest R package ^28^. Standard error of the difference was derived from the predictmeans package in R ^29^ for each linear mixed effect model. For comparisons between antibodies AMA1 (3D7, L32) and MSP1 (FVO) the sample sizes for each group and year were: Continuous 11, Plateau 6 (2015); Continuous 10, Plateau 8 (2016) and; Continuous 10, Plateau 8 (2017). The comparison group sizes for antibodies AMA1 (FVO), MSP1 (3D7, FUP) and MSP3 (FVO) were: Continuous 10, Plateau 8 (2015); Continuous 10, Plateau 8 (2016) and; Continuous 10. Plateau 8 (2017).

## Results

### Large between-child variation in accumulation of clinical episodes over time

**Figure 1.**
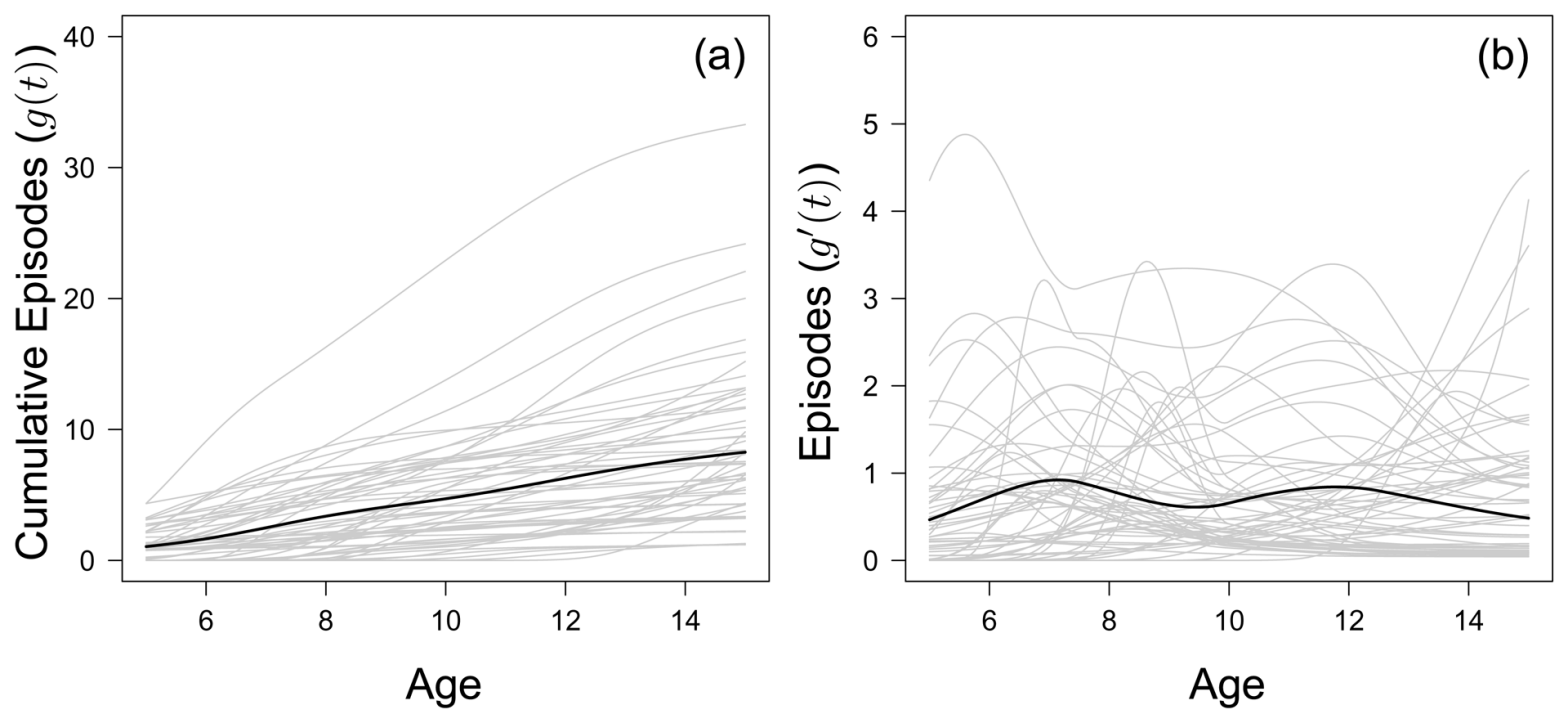
( **a**) The fitted monotonic increasing functions to cumulative malaria episodes against age for all children (grey) who left the study at 15 years of age, with the mean fitted line (black) to all children. ( **b**) The first derivative of the fitted monotonic functions in ( **a**), considered as the number of episodes each child experiences in a year.

Figure 1a shows the fitted accumulated number of clinical malaria episodes of all 56 children born between 2001 and 2003 who completed the cohort study period. The inter-quartile range of cumulative episodes by the age of 15 was 4–11.25, with a median of 7. The range in accumulated malaria episodes was large, with one child accumulating 32 episodes by the age of 15 compared to another child, who experienced only 1 clinical episode before the age of 15. The fitted year-to-year variation in episodes experienced by each child is given in Figure 1.

**Figure 2.**
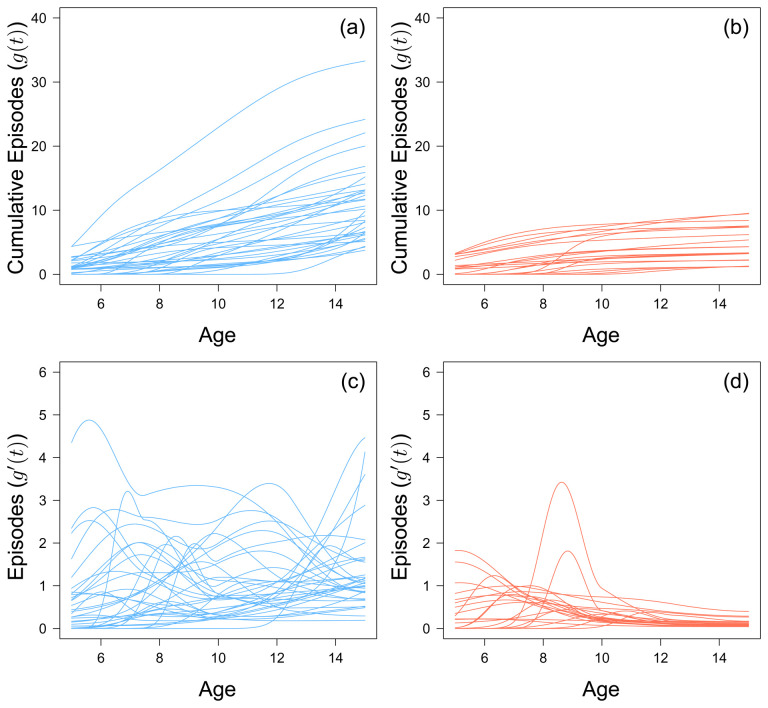
The fitted monotonic increasing functions to cumulative malaria episodes against age for children who experienced a malaria episode between the age of 13 and 15 in blue ( **a**) and plateaued in their episodes at the age of 12 in red ( **b**). The first derivative of the fitted monotonic functions of children who experienced a malaria episode between the age of 13 and 15 in blue ( **c**) and plateaued in their episodes at the age of 12 in red ( **d**).

By the age of 8, 2 out of 56 children do not go on to experience any further clinical malaria episode over the entire study period. This value increases to 22 out of 56 by age 12. Generally, there does not seem to be any discernible trend in terms of cumulative number of episodes for the 38 children who experienced an episode within the last three years of the study ( Figure 2a, c). Of the 22 children who stop experiencing episodes before the last three years, the rate at which they accumulated episodes slowed after an initial peak, but this peak varied for each child ( Figure 2b, d). There does not seem to be a specific age where children as a whole suddenly acquire episodes. However, children who plateaued in their accumulation of clinical malaria episodes did not experience more than 9 episodes.

### Children who stop experiencing clinical episodes experienced a higher parasite density

**Figure 3.**
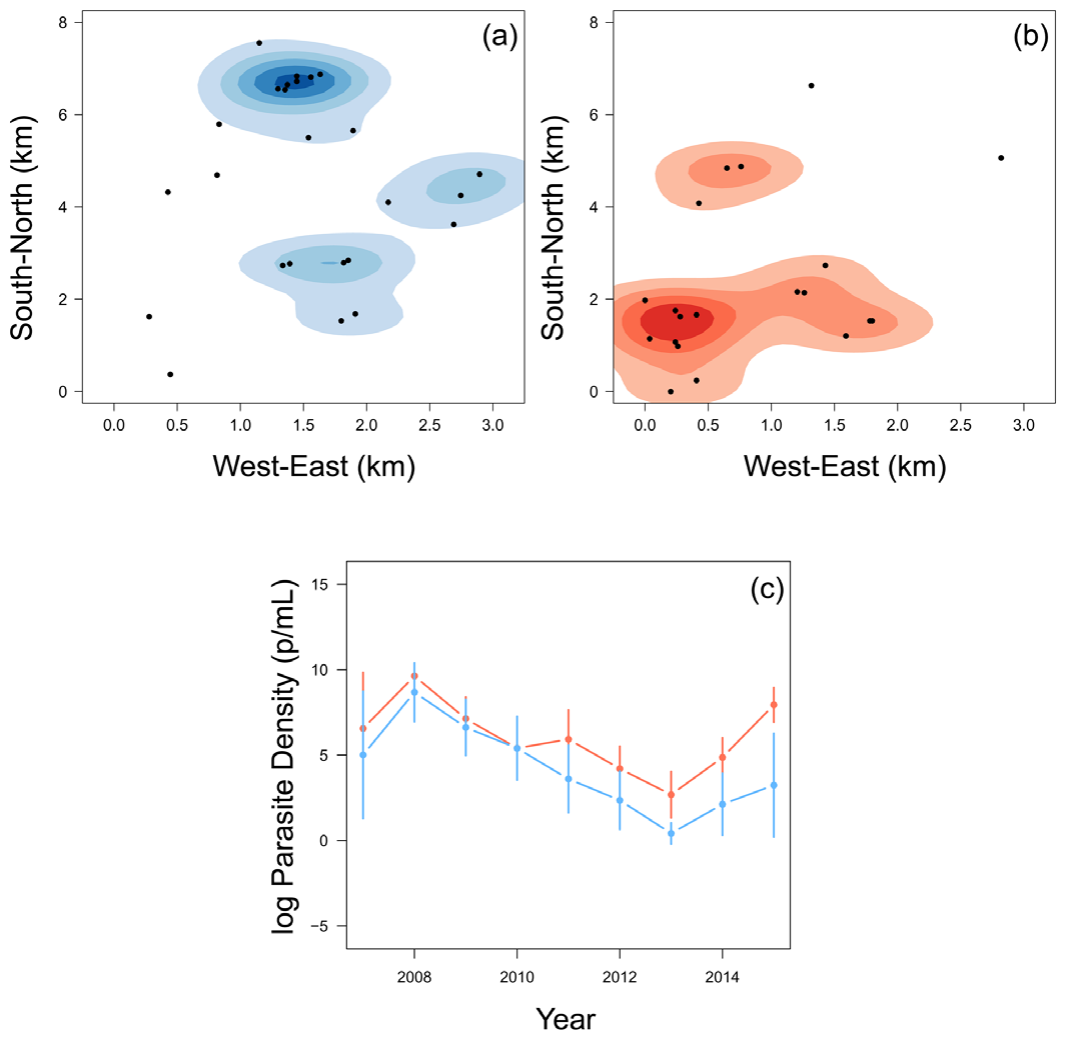
A smoothed histogram of the location of children who experienced a malaria episode between the age of 13 and 15 in blue ( **a**) and plateaued in their episodes at the age of 12 in red ( **b**). The mean (+-95% confidence intervals) of the log parasite density of the annual cross-sectional survey for children who experienced a malaria episode between the age of 13 and 15 in blue and plateaued in their episodes by the age of 12 in red ( **c**).

The 22 children who stopped experiencing episodes in the last few years of the study tended to be in the South-West region of the region, whereas the rest of the children were mostly located in the North-East region ( Figure 3a–b). When considering their annual asymptomatic (cross-sectional) parasite densities (parasite/mL) of children who were parasite positive, there was little difference until 2010. Of the children who were found to be parasite positive at the time of asymptomatic infection, from 2010, the children who then stopped experiencing episodes had, on average, higher asymptomatic parasite densities than other children ( Figure 3c). This finding is in agreement with the assumption that ability to carry higher parasitemia and remain asymptomatic is in fact a product of immunity. 2015 was the year with the largest difference and incidentally marked the period when most children within this group experienced their final clinical episode.

### Chidren who stop experiencing clinical episodes are characterized by higher levels of circulating malaria-specific antibodies

Children who plateaued in their accumulation of clinical episodes had higher levels of antibodies, specific for a number of key *Pf* antigens compared to children who continuously experienced clinical malaria episodes (AMA1 (3D7 ( *F*_1,16_ = 6.81, *p* = 0.019), FVO ( *F*_1,16_ = 7.77, *p* = 0.013), L32 ( *F*_1,16.01_ = 7.11, *p* = 0.017) and MSP3 (FVO ( *F*_1,16_ = 22.65, *p <* 0.001); Table 1). MSP1 was the only antigen for which there were no distinct differences between the two groups of children ( Figure 5).

**Table 1.** The Analysis of Variance tables for each antibody response at the Group, Year and Group:Year level, with corresponding F-tests and p-values.

AMA1: 3D7	Sum Square	Mean Square Error	Numerator DF	Denominator DF	F value	p-value
Group	1.43	1.43	1	16	6.81	0.019
Year	6.39	3.20	2	31.11	15.26	<0.001
Group:Year	0.25	0.13	2	31.12	0.61	0.552
AMA1: FVO	Sum Square	Mean Square Error	Numerator DF	Denominator DF	F value	p-value
Group	1.82	1.82	1	16	7.77	0.013
Year	5.99	2.99	2	32	12.80	<0.001
Group:Year	1.40	0.70	2	32	3.00	0.064
AMA1: L32	Sum Square	Mean Square Error	Numerator DF	Denominator DF	F value	p-value
Group	1.93	1.93	1	16.01	7.11	0.017
Year	6.79	3.39	2	31.15	12.51	<0.001
Group:Year	0.14	0.07	2	31.17	0.26	0.776
MSP1: 3D7	Sum Square	Mean Square Error	Numerator DF	Denominator DF	F value	p-value
Group	2.09	2.09	1	16	3.03	0.101
Year	7.35	3.68	2	32	5.34	0.010
Group:Year	0.94	0.47	2	32	0.68	0.513
MSP1: FUP	Sum Square	Mean Square Error	Numerator DF	Denominator DF	F value	p-value
Group	0.92	0.92	1	16	2.58	0.128
Year	13.59	6.80	2	32	19.04	<0.001
Group:Year	0.38	0.19	2	32	0.53	0.596
MSP1: FVO	Sum Square	Mean Square Error	Numerator DF	Denominator DF	F value	p-value
Group	0.03	0.03	1	15.92	0.15	0.702
Year	6.44	3.22	2	32.53	16.75	<0.001
Group:Year	0.06	0.03	2	32.61	0.15	0.858
MSP3: FVO	Sum Square	Mean Square Error	Numerator DF	Denominator DF	F value	p-value
Group	4.68	4.68	1	16	22.65	<0.001
Year	5.88	2.94	2	32	14.22	<0.001
Group:Year	0.50	0.25	2	32	1.21	0.312

**Figure 4.**
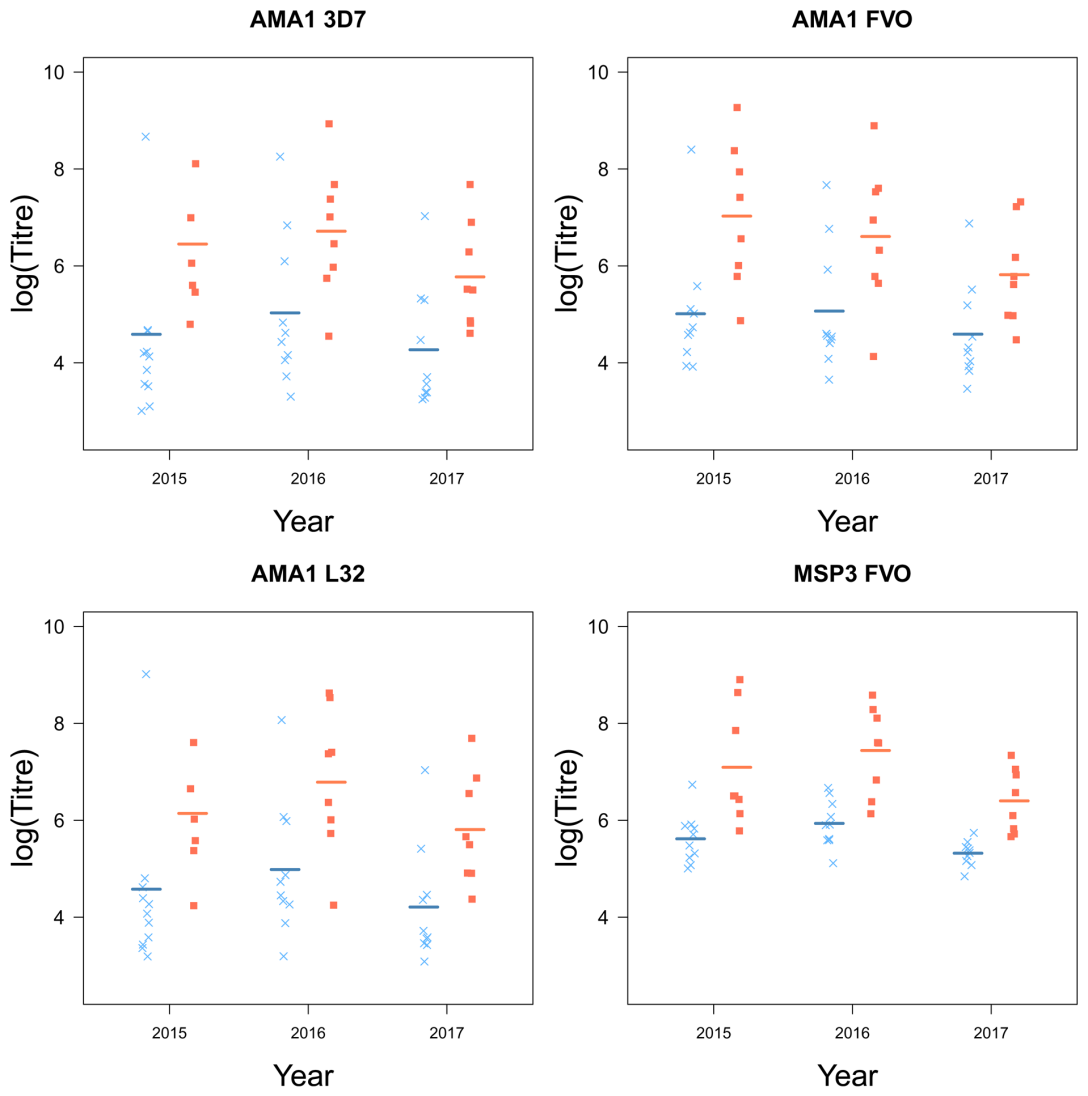
The antigens AMA1 and MSP3 and their strains from children who experienced a malaria episode between the age of 13 and 15 (blue *×*) and plateaued in their episodes at the age of 12 (red ■) from 2015 to 2017, with fitted values (line) from the linear mixed model.

**Figure 5.**
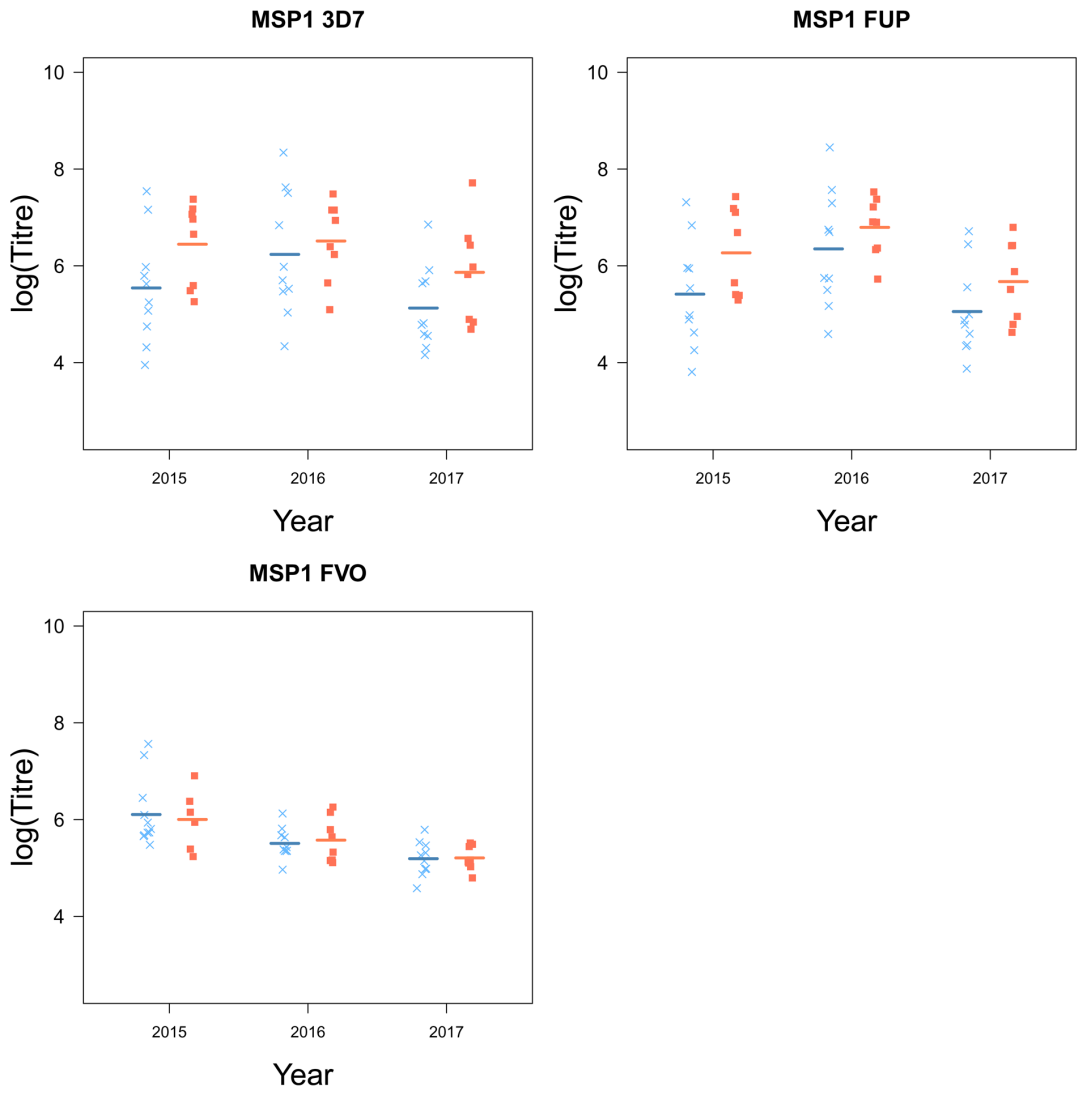
The antigen MSP1 and their strain from children who experienced a malaria episode between the age of 13 and 15 (blue *×*) and plateaued in their episodes at the age of 12 (red ■) from 2015 to 2018).

Further, there were large yearly differences across all groups in the levels of circulating antibodies (AMA1 (3D7 ( *F*_2,31.11_ = 15.26, *p <* 0.001), FVO ( *F*_2,32_ = 12.80, *p <* 0.001), L32 ( *F*_2,31.15_ = 12.51, *p <* 0.001), MSP1 (3D7 ( *F*_2,32_ = 5.34, *p* = 0.010), FUP ( *F*_2,32_ = 19.04, *p <* 0.001), FVO ( *F*_2,32.53_ = 16.75, *p <* 0.001) and MSP3 FVO ( *F*_2,32_ = 14.22, *p <* 0.001), Table 1). However, the year effect of antibody production was consistent across all antibodies specificities ( Figure 4 and Figure 5).

## Discussion

From this 10-year observational study, our results demonstrate that small changes in geographic location can impact the accumulation of clinical manifestations of malaria. Children who continued to have episodes throughout the study were generally located in the North-East part of the study area and tended to be characterised by lower asymptomatic parasite densities and lower levels of circulating *Pf* -specific antibodies. These results indicate that micro-geographic regions of high parasite exposure ^30^ have an impact on the acquisition of immunity, where children from the same sub-region develop immunity at different rates based on their exposure to the parasite.

Human cohort studies provide a unique opportunity to investigate the development of immunity to malaria. However interpreting such studies is often a challenge as using number of clinical episodes as a measure of immunity makes it difficult to distinguish between immune individuals and those who are simply less exposed to the parasite. In this study, we analyzed data from a ten-year longitudinal cohort of children using growth curves to capture the heterogeneity of accumulated clinical episodes, allowing for a better interpretation into more-immune and lessimmune individuals. From these curves, large variations in the rate of accumulation of clinical episodes were observed, illustrating the challenges associated with extrapolating from such data to investigate the development of immunity to malaria.

Two sub-populations of children were identified; children who plateaued in the accumulation of clinical episodes at or before the age of 12, and those children who continued to experience clinical episodes between the age of 13 and 15. Those children who plateaued in their accumulation of malaria episodes and who were infected at the time of asymptomatic sampling had, on average, higher asymptomatic parasite densities of *Pf* and were generally located in the South-West region of Junju ( Figure 3c). Furthermore, children who plateaued had higher levels of circulating malaria-specific antibodies AMA1 and MSP3 ( Figure 4). The regional differences in accumulated episodes appear to agree with our findings of spatial differences in the prevalence of clinical malaria among this cohort, with children in the South-West experiencing fewer clinical episodes ^31^. Our results show these regional differences seem to be reflected in the development of protective immunity.

Our findings agree with previous data, which suggest that protection from clinical malaria is associated with higher titres of *Pf* -specific antibodies ^32^ as well as an ability to remain asymptomatic whilst carrying higher parasite densities ^33^. The reducing rate of accumulation of clinical episodes with age is indicative of developing anti-disease immunity, i.e., the ability to tolerate higher parasite densities without clinical malaria. This could be the result of higher exposure to *Pf* in the micro-environment of South-West Junju. It is intriguing that these higher parasite densities are maintained despite the higher levels of anti-AMA1 and MSP3 antibodies in the plateauing group. This suggests that these antibodies are not contributing significantly to anti-parasite immunity but are rather a reflection of the level of *Pf* infection.

Longitudinal surveillance cohorts are a very powerful tools to study anti-malarial immunity and a growing number of studies are adopting such a design in exploring the immune mechanisms responsible for mediating such immunity ^6, 34, 35^. These studies often classify individuals within their cohorts as immune or non-immune based on the total accumulated numbers of episodes that an individual has experienced over a period of time. Given the heterogeneous spatial and temporal distribution of the malaria parasite within a geographic area and study period respectively, such an approach is likely to be confounded by variations in exposure. By assessing each study participant’s malaria history over ten years, we were able to provide a more comprehensive analysis of the diversity of malaria history within a cohort, facilitating more accurate identification of individual immune status and ultimately a less confounded investigation of the mechanisms responsible for development of partial immunity to malaria.

## Data availability

### Underlying data

Harvard Dataverse: Replication Data for: 10-year longitudinal study of malaria in children: Insights into acquisition and maintenance of naturally acquired immunity. https://doi.org/10.7910/DVN/X4L47D ^36^.

This project includes the following underlying data:

- Epi_Data_Pub.tab (underlying data file)
- JAddy_Epi_Data_Codebook.pdf (data code book)
- SummaryofAnalysis.html (R analysis script)

Data are available under the terms of the Creative Commons Attribution 4.0 International license (CC-BY 4.0).
