# Supplementary material for: 10-year longitudinal study of malaria in children: Insights into acquisition and maintenance of naturally acquired immunity
Source: Wellcome Open Res. 2022 Feb 4;6:79. Originally published 2021 Apr 9. [Version 3] doi: 10.12688/wellcomeopenres.16562.3 (PMC8822141; doi:10.12688/wellcomeopenres.16562.3)
Supplement: Peer review history

# Peer review: Reviewer response for version 3

**DOI:** 10.21956/wellcomeopenres.19559.r48528

**Published:** 2022-02-07

**Authors:** Rhea J Longley

**Affiliations:** 1 Population Health and Immunity Division, Walter and Eliza Hall Institute of Medical Research, University of Melbourne, Melbourne, Victoria, Australia

My concern has been addressed.

Is the work clearly and accurately presented and does it cite the current literature?

Partly

If applicable, is the statistical analysis and its interpretation appropriate?

Yes

Are all the source data underlying the results available to ensure full reproducibility?

Yes

Is the study design appropriate and is the work technically sound?

Yes

Are the conclusions drawn adequately supported by the results?

Yes

Are sufficient details of methods and analysis provided to allow replication by others?

Partly

Reviewer Expertise:

Immunology of infectious diseases

I confirm that I have read this submission and believe that I have an appropriate level of expertise to confirm that it is of an acceptable scientific standard.

# Peer review: Reviewer response for version 2

**DOI:** 10.21956/wellcomeopenres.19279.r47420

**Published:** 2021-12-17

**Authors:** Rhea J Longley

**Affiliations:** 1 Population Health and Immunity Division, Walter and Eliza Hall Institute of Medical Research, University of Melbourne, Melbourne, Victoria, Australia

Thank you to the authors for addressing my comments. The updated manuscript communicates the research very nicely. I particularly appreciate the updated description of the study design and inclusion of Table 1.

 I am still somewhat confused by the response to my question on the ELISA and use of mixed alleles. I understand the concept behind wanting to mix alleles, but the results (in the text, table and figures) are presented per allele i.e. 3D7 and FVO separately. So were the antigen alleles mixed 1:1 and also assessed individually in the ELISA? Thank you for the clarification.

Is the work clearly and accurately presented and does it cite the current literature?

Partly

If applicable, is the statistical analysis and its interpretation appropriate?

Yes

Are all the source data underlying the results available to ensure full reproducibility?

Yes

Is the study design appropriate and is the work technically sound?

Yes

Are the conclusions drawn adequately supported by the results?

Yes

Are sufficient details of methods and analysis provided to allow replication by others?

Partly

Reviewer Expertise:

Immunology of infectious diseases

I confirm that I have read this submission and believe that I have an appropriate level of expertise to confirm that it is of an acceptable scientific standard, however I have significant reservations, as outlined above.

# Response

**Published:** 2022-01-30

**Authors:** John Addy

**Affiliations:** Francis Crick Institute, UK

We thank the reviewer for their response.

 This part of the methods has been corrected. The sentence " *For AMA1, ELISA plates were coated with a 1:1 mixture of FVO and 3D7 alleles"* was included in error.

# Peer review: Reviewer response for version 2

**DOI:** 10.21956/wellcomeopenres.19279.r47421

**Published:** 2021-12-13

**Authors:** Rodrigo M Corder

**Affiliations:** 1 University of California, Berkeley School of Public Health, Berkeley, CA, USA

I thank the authors for their consideration of my comments. The additional information they provide in the publication satisfies my earlier concerns.

Is the work clearly and accurately presented and does it cite the current literature?

Partly

If applicable, is the statistical analysis and its interpretation appropriate?

Yes

Are all the source data underlying the results available to ensure full reproducibility?

Yes

Is the study design appropriate and is the work technically sound?

Yes

Are the conclusions drawn adequately supported by the results?

Yes

Are sufficient details of methods and analysis provided to allow replication by others?

Yes

Reviewer Expertise:

Mathematical modelling of infectious diseases

I confirm that I have read this submission and believe that I have an appropriate level of expertise to confirm that it is of an acceptable scientific standard.

# Peer review: Reviewer response for version 1

**DOI:** 10.21956/wellcomeopenres.18251.r45152

**Published:** 2021-09-06

**Authors:** Rhea J Longley

**Affiliations:** 1 Population Health and Immunity Division, Walter and Eliza Hall Institute of Medical Research, University of Melbourne, Melbourne, Victoria, Australia

This manuscript studies the acquisition of clinical episodes of malaria over 10 years and identifies two patterns; one where children stop acquiring further clinical episodes after age 12 and one where they continue to acquire them. They relate this to both asymptomatic parasite densities and anti-malarial antibody responses. The data availability is fantastic and very clear with the additional files outlining the variable information and the script used for analyses. The manuscript is generally well-written but could use further clarification on the points below (in addition to points made by the first reviewer).

 Major points:

1. Data from the yearly cross-sectional surveys: the authors present asymptomatic parasite densities in Fig3c but as far as I can tell, they have not presented a summary of this data i.e. # of children with asymptomatic infections at each yearly survey and thus # of children that make up the data presented in Fig3c. I think this is important for their conclusion “children from the same sub-region develop immunity at different rates based on their exposure to the parasite”. Are the authors relating exposure to the parasite density or the total # infections (inc asymptomatic) experienced? Are all 56 children infected at each survey (I think no from the dataset)? Are the data in Fig3c from the blood-smears or qPCR (qPCR I believe, but methods states blood smear densities are used in the paper)?
2. ELISA results: in the methods it said the two alleles for AMA1 were mixed, so I am unclear how there are different results presented for both alleles? As the authors state in the introduction, current infections can have a big impact on antibody levels – can they comment on whether individuals from either group had current infections at the time the sampling was made for antibody measurements? In those that have current infections have the authors directly correlated/associated the parasite density with antibody level i.e. to give strength to their statement that antibodies are “a reflection of the level of Pf infection”?

Minor points:

1. Study population: This section in the methods is a little difficult to follow given it’s a rolling enrolment and then a sub-group has been used. The 2 ^nd^ paragraph in particular could be revised so it covers the whole cohort first then describes the 56 kids in this study. Would a schematic help? This might also help clarify the sampling for the yearly surveys, clinical episodes, antibodies etc.
2. Antigen/ELISA technical details: antigens need more info i.e. the expression system and construct – this could be referenced to a prior publication (it seems the references listed more relate to the statement about associations with protection, but the authors could clarify). What hyperimmune sample/s were used for the standard curve? Are they available for others to use i.e. is this a reference pool? How was the standard curve conversion performed? If this has all been done before a reference can be cited.
3. Fig3C: the parasite densities between the two groups are still fairly similar and do vary year on year - can the authors comment on whether that difference in parasite density is meaningful/statistically significant?
4. Discussion on characterising exposure in longitudinal cohorts: how about work that uses genotyping to understand the force of infection (PMID: 22665809 ^1^)/molecular force of blood-stage infection (PMID: 24040428 ^2^)? Or antibodies against mosquito salivary gland antigens (PMID: 21175067 ^3^, PMID: 22195000 ^4^)?
5. Final sentence in discussion on mechanisms responsible for partial immunity “ *By assessing each study participant’s malaria history over ten years, we were able to provide a more comprehensive analysis of the diversity of malaria history within a cohort, facilitating more accurate identification of individual immune status and ultimately a less confounded investigation of the mechanisms responsible for development of partial immunity to malaria*”. It might be worth also reflecting on work by others suggesting functional antibody assays are needed to identify targets of immunity rather than magnitude alone (PMID: 30723225 ^5^), as it could be argued the current manuscript does not identify the targets or mechanisms of the acquired immunity in these children – scope for future work.

I cannot comment on technical details of the mathematical models.

Is the work clearly and accurately presented and does it cite the current literature?

Partly

If applicable, is the statistical analysis and its interpretation appropriate?

Yes

Are all the source data underlying the results available to ensure full reproducibility?

Yes

Is the study design appropriate and is the work technically sound?

Yes

Are the conclusions drawn adequately supported by the results?

Yes

Are sufficient details of methods and analysis provided to allow replication by others?

Partly

Reviewer Expertise:

Immunology of infectious diseases

I confirm that I have read this submission and believe that I have an appropriate level of expertise to confirm that it is of an acceptable scientific standard, however I have significant reservations, as outlined above.

## References

[1] : Force of infection is key to understanding the epidemiology of Plasmodium falciparum malaria in Papua New Guinean children. *Proc Natl Acad Sci U S A* .2012;109(25) : 10.1073/pnas.1200841109 10030-5 10.1073/pnas.1200841109 22665809PMC3382533

[2] : A high force of plasmodium vivax blood-stage infection drives the rapid acquisition of immunity in papua new guinean children. *PLoS Negl Trop Dis* .2013;7(9) : 10.1371/journal.pntd.0002403 e2403 10.1371/journal.pntd.0002403 24040428PMC3764149

[3] : Antibody response against Anopheles albimanus (Diptera: Culicidae) salivary protein as a measure of mosquito bite exposure in Haiti. *J Med Entomol* .2010;47(6) : 10.1603/me09240 1156-63 10.1603/me09240 21175067

[4] : Relationship between exposure to vector bites and antibody responses to mosquito salivary gland extracts. *PLoS One* .2011;6(12) : 10.1371/journal.pone.0029107 e29107 10.1371/journal.pone.0029107 22195000PMC3237593

[5] : Targets of complement-fixing antibodies in protective immunity against malaria in children. *Nature Communications* .2019;10(1) : 10.1038/s41467-019-08528-z 10.1038/s41467-019-08528-z PMC636379830723225

# Response

**Published:** 2021-11-19

**Authors:** John Addy

**Affiliations:** Francis Crick Institute, UK

We thank both reviewers for the positive review of our work. Their comments have added to the manuscript and have improved the quality and readability of the manuscript. We have addressed each point with and answer. In some cases, we have provided more information and clarity, in other cases we have accepted the issue and added to our manuscript accordingly. Responses from all reviewers have been included here.

*This manuscript studies the acquisition of clinical episodes of malaria over 10 years and identifies two patterns; one where children stop acquiring further clinical episodes after age 12 and one where they continue to acquire them. They relate this to both asymptomatic parasite densities and anti-malarial antibody responses. The data availability is fantastic and very clear with the additional files outlining the variable information and the script used for analyses. The manuscript is generally well-written but could use further clarification on the points below (in addition to points made by the first reviewer).*

**

*Major points:*

*Data from the yearly cross-sectional surveys: the authors present asymptomatic parasite densities in Fig3c but as far as I can tell, they have not presented a summary of this data i.e. # of children with asymptomatic infections at each yearly survey and thus # of children that make up the data presented in Fig3c. I think this is important for their conclusion “children from the same sub-region develop immunity at different rates based on their exposure to the parasite”. Are the authors relating exposure to the parasite density or the total # infections (inc asymptomatic) experienced? Are all 56 children infected at each survey (I think no from the dataset)? Are the data in Fig3c from the blood-smears or qPCR (qPCR I believe, but methods states blood smear densities are used in the paper)?*

 Added new Table 1 about yearly summaries cross-sectional surveys which answers this point and addressed in text (see Section Children who stop experiencing clinical episodes experienced a higher parasite density). Exposure to the parasite is assessed using both asymptomatic measurements and clinical episodes. All 56 children live in a malaria region. qPCR data were used for Figure 3c.

*ELISA results: in the methods it said the two alleles for AMA1 were mixed, so I am unclear how there are different results presented for both alleles? As the authors state in the introduction, current infections can have a big impact on antibody levels – can they comment on whether individuals from either group had current infections at the time the sampling was made for antibody measurements? In those that have current infections have the authors directly correlated/associated the parasite density with antibody level i.e. to give strength to their statement that antibodies are “a reflection of the level of Pf infection”?*

 The mixing of the two alleles means that we are only interested in the antibody response to AMA1 – irrespective to whatever alleles. Mixing ensures we minimise missing the response owing to the polymorphism. There are too few samples to do an adequate correlation between parasite density for a few of the antibodies. Added reference of evidence of relationship between parasite and antibodies in text (See the Discussion).

*Minor points:*

*Study population: This section in the methods is a little difficult to follow given it’s a rolling enrolment and then a sub-group has been used. The 2nd paragraph in particular could be revised so it covers the whole cohort first then describes the 56 kids in this study. Would a schematic help? This might also help clarify the sampling for the yearly surveys, clinical episodes, antibodies etc.*

 This question is similar to the other reviewers question, see the response to the other reviewer.

*Antigen/ELISA technical details: antigens need more info i.e. the expression system and construct – this could be referenced to a prior publication (it seems the references listed more relate to the statement about associations with protection, but the authors could clarify). What hyperimmune sample/s were used for the standard curve? Are they available for others to use i.e. is this a reference pool? How was the standard curve conversion performed? If this has all been done before a reference can be cited.*

 This has been addressed in text (see Methods section “Antigens for ELISA”).

**

*Fig3C: the parasite densities between the two groups are still fairly similar and do vary year on year - can the authors comment on whether that difference in parasite density is meaningful/statistically significant?*

 This difference is on the log-scale and shows that the parasite density is similar in both groups until 2010. This difference illustrates that although the temporal trends look similar, there is obvious spatial effects, plateauers seem to have higher parasite densities since 2010, but tend to live in the South-West. We cannot say which parasite density cut-off is responsible for the development of immunity here. A formal statistical comparison here is would not provide additional information as 95% confidence intervals have been produced.

*Discussion on characterising exposure in longitudinal cohorts: how about work that uses genotyping to understand the force of infection (PMID: 226658091)/molecular force of blood-stage infection (PMID: 240404282)? Or antibodies against mosquito salivary gland antigens (PMID: 211750673,* *PMID: 221950004)?*

 Changed in text (see Discussion).

**

*Final sentence in discussion on mechanisms responsible for partial immunity “By assessing each study participant’s malaria history over ten years, we were able to provide a more comprehensive analysis of the diversity of malaria history within a cohort, facilitating more accurate identification of individual immune status and ultimately a less confounded investigation of the mechanisms responsible for development of partial immunity to malaria”. It might be worth also reflecting on work by others suggesting functional antibody assays are needed to identify targets of immunity rather than magnitude alone (PMID: 307232255), as it could be argued the current manuscript does not identify the targets or mechanisms of the acquired immunity in these children – scope for future work.*

*I cannot comment on technical details of the mathematical models.*

 Changed in text (see Discussion).

# Peer review: Reviewer response for version 1

**DOI:** 10.21956/wellcomeopenres.18251.r45029

**Published:** 2021-07-22

**Authors:** Rodrigo M Corder

**Affiliations:** 1 University of California, Berkeley School of Public Health, Berkeley, CA, USA

This manuscript studies the variation in the number of clinical episodes experienced by children of the same age within the same cohort and relates it to the development of naturally acquired immunity. The analysis presented is particularly important as it highlights the need to account for heterogeneity within endemic populations in order to better define control and elimination measures. This is a well written manuscript and I have a few comments:

  

1. If antibody levels were compared between plateauers and children who experienced episodes up to the last three years of the cohort study, it is not clear to me why the sample sizes are different between comparisons AMA1(3D7, L32)/MSP1(FVO) and AMA1(FVO)/MSP1(3D7,FUP)/MSP3(FVO) over the years. For example, considering 2015, “continuous” are 11 when the comparison is between antibodies AMA1 (3D7, L32) and MSP1 (FVO), and 10 when comparison is between antibodies AMA1 (FVO), MSP1 (3D7, FUP) and MSP3 (FVO). Also, individuals do not add up to 56 (sample size). Please, clarify it.
2. Sentences like “cumulative episodes by the age of 15”, “with one child accumulating 32 episodes by the age of 15”, “another child, who experienced only 1 clinical episode before the age of 15” and “children who plateaued in their accumulation of clinical malaria episodes did not experience more than 9 episodes” are technically inconsistent if the analysis comprised children aged from 5 to 15. Please, rephrase them. The authors should also consider starting Figure x-tick labels (Age) with 5 and ending it with 15.
3. If my understanding is correct, by selecting 56 children who were born between 2001 and 2003, the age of them should not be equal at a given time. Therefore, why do the authors choose to do antibody analysis over “Year” (Figures 3c, 4 and 5 and Table 1) and not over “Age” (like in Figures 1 and 2)? It seems to me that the analysis over “Age” is one that best assesses each study participant's history of malaria. Please, clarify it.
4. The sentence “MSP1 was the only antigen for which there were no distinct differences between the two groups of children” may lead to wrong inferences. The authors should consider mentioning the sample size of the analysis as one of its limitations and improve the discussion about MSP1 antigen results.

I cannot comment on technical details of antibody methods and techniques.

Is the work clearly and accurately presented and does it cite the current literature?

Partly

If applicable, is the statistical analysis and its interpretation appropriate?

Yes

Are all the source data underlying the results available to ensure full reproducibility?

Yes

Is the study design appropriate and is the work technically sound?

Yes

Are the conclusions drawn adequately supported by the results?

Yes

Are sufficient details of methods and analysis provided to allow replication by others?

Yes

Reviewer Expertise:

Mathematical modelling of infectious diseases

I confirm that I have read this submission and believe that I have an appropriate level of expertise to confirm that it is of an acceptable scientific standard, however I have significant reservations, as outlined above.

# Response

**Published:** 2021-11-19

**Authors:** John Addy

**Affiliations:** Francis Crick Institute, UK

*This manuscript studies the variation in the number of clinical episodes experienced by children of the same age within the same cohort and relates it to the development of naturally acquired immunity. The analysis presented is particularly important as it highlights the need to account for heterogeneity within endemic populations in order to better define control and elimination measures. This is a well written manuscript and I have a few comments:*

**

*If antibody levels were compared between plateauers and children who experienced episodes up to the last three years of the cohort study, it is not clear to me why the sample sizes are different between comparisons AMA1(3D7, L32)/MSP1(FVO) and AMA1(FVO)/MSP1(3D7,FUP)/MSP3(FVO) over the years. For example, considering 2015, “continuous” are 11 when the comparison is between antibodies AMA1 (3D7, L32) and MSP1 (FVO), and 10 when comparison is between antibodies AMA1 (FVO), MSP1 (3D7, FUP) and MSP3 (FVO). Also, individuals do not add up to 56 (sample size). Please, clarify it.*

 The initial part of this study focuses on children who have completed the cohort study and left when they were 15 by 2018 (so 10  years’ worth of data for each individual, 56 in total, some children completed the cohort earlier, 2016 or 2015 for example). When comparing antibody data we want to remove all possible confounders, such as age. Also, the antibody data follow a repeated measures design, the same children sampled each year. Further, there is strong year-to-year variation in clinical malaria episodes and parasite densities across the study. So, in order to obtain an unbiased antibody comparison between plateauers and non-plateauers children who have antibody measurements across all three years were considered, and these were all children of the same age. A linear-mixed-model (LMM) was used, to identify the individual effect of each child and this was modelled as a random effect. The reason why 11 children were included for some antibodies and 10 for other, is because these are the same individuals, but there was a missing value for AMA1 (FVO), MSP1 (3D7, FUP) and MSP3 (FVO) antibodies. We reduced the influence this missing value has on our result by using a linear-mixed-model (LMM) and having child as a random effect. This has been clarified in the text (see Statistical Analysis section).

*Sentences like “cumulative episodes by the age of 15”, “with one child accumulating 32 episodes by the age of 15”, “another child, who experienced only 1 clinical episode before the age of 15” and “children who plateaued in their accumulation of clinical malaria episodes did not experience more than 9 episodes” are technically inconsistent if the analysis comprised children aged from 5 to 15. Please, rephrase them. The authors should also consider starting Figure x-tick labels (Age) with 5 and ending it with 15.*

 Changed in text (see “Large between-child variation in accumulation of clinical episodes over time” section).

*If my understanding is correct, by selecting 56 children who were born between 2001 and 2003, the age of them should not be equal at a given time. Therefore, why do the authors choose to do antibody analysis over “Year” (Figures 3c, 4 and 5 and Table 1) and not over “Age” (like in Figures 1 and 2)? It seems to me that the analysis over “Age” is one that best assesses each study participant's history of malaria. Please, clarify it.*

 Following from above, to ensure Age was not confounding our plateauers vs non-plateauers comparison, only children of the same age and over three years were considered. We could not adequately do an Age comparison due to the oldest children leaving the study after one antibody sample. Therefore, any Age comparison (only data from 2015) would be confounded by 2015 being a high transmission year. Age is not a variable of interest in the manuscript but rather whether children plateau or not in their clinical symptoms. As this illustrates the complexity of longitudinal malaria cohort studies.

*The sentence “MSP1 was the only antigen for which there were no distinct differences between the two groups of children” may lead to wrong inferences. The authors should consider mentioning the sample size of the analysis as one of its limitations and improve the discussion about MSP1 antigen results.*

 Changed in text (see “Children who stop experiencing clinical episodes are characterized by higher levels of circulating malaria-specific antibodies” section).
